# MAPK/ERK and JNK pathways regulate lipid synthesis and cell growth of *Chlamydomonas reinhardtii* under osmotic stress, respectively

**DOI:** 10.1038/s41598-018-32216-5

**Published:** 2018-09-14

**Authors:** Ahreum Yang, William I. Suh, Nam Kyu Kang, Bongsoo Lee, Yong Keun Chang

**Affiliations:** 1 Department of Chemical and Biomolecular Engineering, KAIST, 291 Daehak-ro, Yuseong-gu, Daejeon 305-701 Republic of Korea; 2 Advanced Biomass R&D Center, KAIST, 291 Daehak-ro, Yuseong-gu, Daejeon 305-701 Republic of Korea

## Abstract

Microalgae have great potential for the production of biofuels due to the ability of the organism to accumulate large quantities of storage lipids under stress conditions. Mitogen activated protein kinase (MAPK) signaling cascades are widely recognized for their role in stress response signal transduction in eukaryotes. To assess the correlation between MAPK activation and lipid productivity, *Chlamydomonas reinhardtii* was studied under various concentrations of NaCl. The results demonstrated that *C*. *reinhardtii* exhibits elevated levels of extracellular-signal regulated kinase (ERK) and c-Jun N-terminal kinase (JNK) activities after undergoing osmotic stress, as well as an increase in cellular lipid content. To establish a more direct causal link between both kinases and lipid productivity, *C*. *reinhardtii* was subjected to biochemically induced regulation of ERK and JNK pathways. Activating the MEK-ERK pathway via C6 ceramide treatment increased ERK activation and lipid production simultaneously, while PD98059 mediated inhibition of the pathway yielded opposite results. Interestingly, suppression of the JNK pathway with SP600125 resulted in a substantial decrease in cell viability under osmotic stress. These results suggest that ERK and JNK MAP kinases have important roles in microalgal lipid accumulation and cell growth under osmotic stress, respectively.

## Introduction

Concerns regarding unsustainable consumption of fossil based energy sources as well as the inevitably dwindling reserves have resulted in an increasing interest in renewable fuel sources. For many decades, biofuels have been proposed as a viable replacement for petroleum, and today up to 4% or the total gasoline consumed in the US consist of corn based ethanol^1^. However, current human agricultural practice is unsustainable for even human food production let alone biofuel production, and thus, there have been increasing shifts in research trends towards the cultivation of unicellular photosynthetic organisms such as microalgae^2^. The main advantages of microalgae include the ability to achieve a higher biomass containing a higher oil content compared with that of conventional crops^3,4^; additionally, they are ecologically sustainable if wastewater is used as a nutrient source for growth^5^. Also, microalgae can be grown on non-arable land, and therefore does not compete with land for food production. While domesticated terrestrial plants that are grown in modern agriculture have undergone extensive strain selection and selective breeding to develop specific characteristics that meet specific human needs, the same cannot be said for microalgae. As such, genetic engineering using forward and reverse genetic approaches is necessary to uplift traits that are optimal for oil production in wild type microalgal strains. An ideal strain that is suitable for biofuel production should possess both high growth rate and lipid content^6^. However, in most cases, these two traits happen to be mutually exclusive; most proliferating algal cells contain little lipid content, which means that most strains require growth-limiting stress condition for the cells to accumulate high levels of lipids^7^.

Different types of stress conditions are known to enhance lipid productivity across many microalgal species^8–11^. One type of growth limiting stress is nutrient starvation. Deprivation of one or more of the essential growth requirements of microalgae is the most effective way to halt the proliferation of cells in a controlled fashion^12–14^. While limitation in the availability of nitrogen, phosphorus, sulfur, and other micronutrients has been found to enhance lipid productivity among different algal species, nitrogen deprivation is the most frequently characterized among the nutrient stress factors^15^. In addition to nutrient stress, it also has been reported that osmotic stress is also capable of enhancing the lipid contents within algae^16–18^. A number of studies showed that increasing the salinity gradient resulted in an increase in the lipid accumulation within the cells^17^. However, subjecting the microalgae to high salinity conditions results in reductions in both growth and overall biomass productivity^17^.

Even though subjecting the microalgae to a stress environment can greatly improve the overall lipid productivity, such a process requires greater complexity in the cultivation protocols. This is because often times a two-step cultivation process, where the cells must be harvested and then transferred from one environment optimized for growth to another environment which is optimized for lipid accumulation, is necessary^19^. In the case of osmotic stress, a two-stage cultivation would require an additional harvesting step. Simply adding salts to the culture would preclude the reusability of the growth medium, and therefore, this would result in a significant increase in the water footprint^20^. This is not feasible, especially in large scale open outdoor cultivation systems, due to a large amount of energy consumption associated with the harvesting. Therefore, a better understanding of the genetics and molecular mechanisms behind stress induced lipid accumulation is necessary to develop an alternative solution.

In eukaryotes, the mitogen activated protein kinase (MAPK) signaling pathway is responsible for cellular responses to various types of external stimuli including biotic and abiotic stresses^21^. There are three different groups of MAPKs, which are the extracellular-signal regulated kinase (ERK), c-Jun N-terminal kinase (JNK), and p38 MAP kinases^22^. The MAPKs are activated by different types of MAPK kinases (MAP2K), which are in turn activated by MAP2K kinases (MAP3K)^22^. The ERK pathway is well known to be involved in cell cycle regulation in mammals and plants, while JNK pathway is known to regulate cell growth and survival^23–26^. However, the mechanism that triggers lipid biosynthesis under stress conditions is unknown.

In a previous nitrogen deprivation study using the microalga *Chlamydomonas reinhardtii*, nineteen transcripts involving signal transduction, including MAP kinases, were found to be highly activated when the cells were subjected under nitrogen deprivation condition^27^. Most importantly, MAPK and cyclic adenosine monophosphate (CAMP) signaling were concluded to be closely related with neutral lipid accumulation in both *C*. *reinhardtii* and *Chlorella vulgaris*^28^. In addition, while osmotic stress has been known to activate lipid synthesis and MAPK signaling pathways in plants, such effects of osmotic stress have not been reported as of yet in microalgae^29^. In the present study, lipid productivity in the cells that underwent biochemically induced upregulation and downregulation of the MAPK pathway was assessed with the goal of conclusively determining the relationship between MAPK and lipid biosynthesis in salt stress conditions.

## Results

### Enhanced activities of MAP kinases under osmotic stress

In order to verify whether MAPK is activated under osmotic stress, western blot analysis of cultures grown under increasing concentrations of NaCl was done for three different types of MAP kinases, specifically ERK, JNK, and p38. Protein samples from 1.5 million cells in the stationary phase were prepared for western blot analysis ensuring that an equal number of cells was used. Among the three different MAPKs analyzed in the preliminary experiment, ERK and JNK activations were shown to have a positive and negative correlation with the increase in salinity, respectively, while the p38 activity was not affected (Fig. S1).

**Figure 1:**
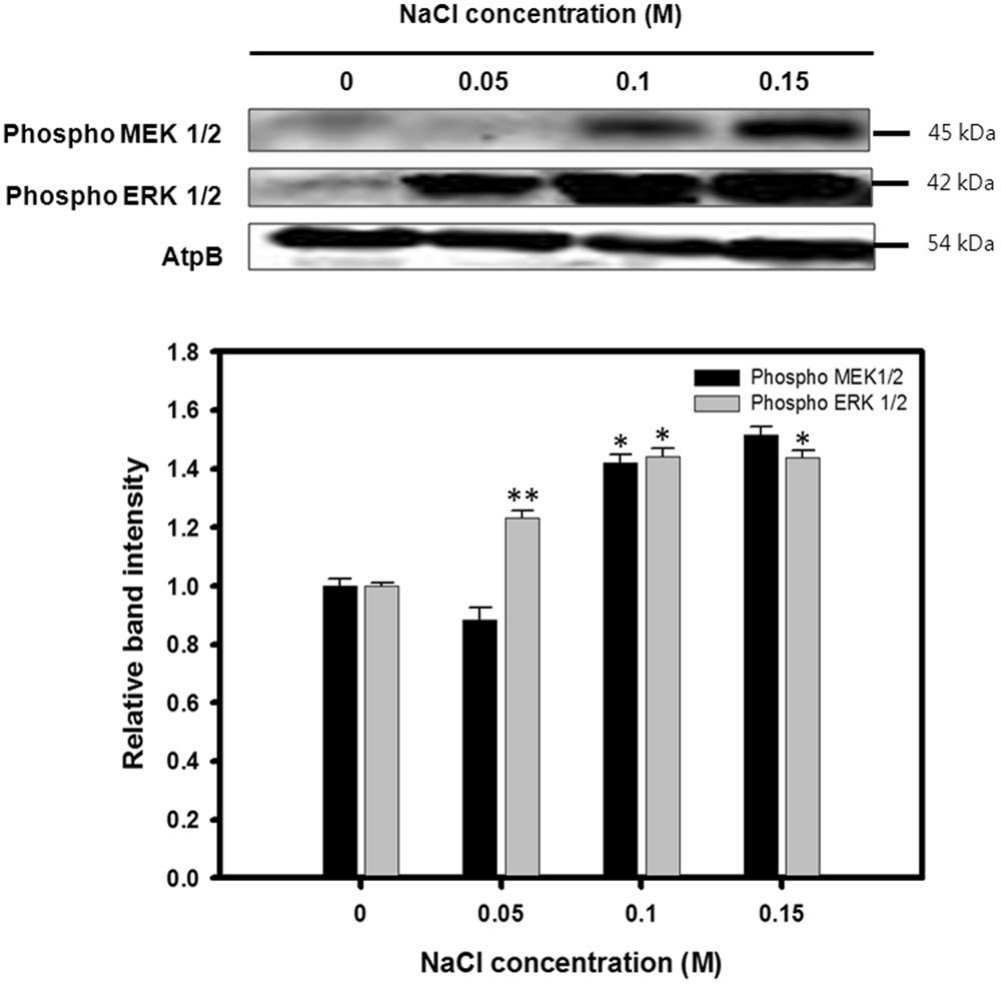
Western blot analysis against anti-MEK1/2 and ERK1/2. ATPβ antibody was detected as a loading control. Cell lysates cultivated under 0, 0.05, 0.1, and 0.15 M concentrations of NaCl were analyzed with phospho-specific antibodies to measure the MEK1/2 and ERK1/2 activities. Full-length blots are presented in Supplementary Fig. S6. Relative expression of proteins is represented as mean ± SD (n = 3). Significant differences were determined by Student’s t-test and indicated by asterisks (*P < 0.05, **P < 0.01, ***P < 0.001).

To further scrutinize how the MEK-ERK pathway is affected by osmotic stress, both the MEK1/2 and ERK1/2 activities were analyzed after the cells were subjected to various levels of osmotic stress for 5 days. The phosphorylation of both MEK1/2 and ERK1/2 was detected with the phospho-specific mammalian MEK1/2 and ERK1/2 antibodies, respectively. The blot revealed bands at the 45 and 42 kD range corresponding to MEK1/2 and ERK1/2, respectively, both of which intensified progressively under increasing levels of osmotic stress (Fig. 1). In terms of the relative band intensities, the ERK activation increased nearly 1.44 fold when the salinity increased from baseline to 0.1 M (Fig. 1).

### Physiological effects of osmotic stress on C. reinhardtii

**Figure 2:**
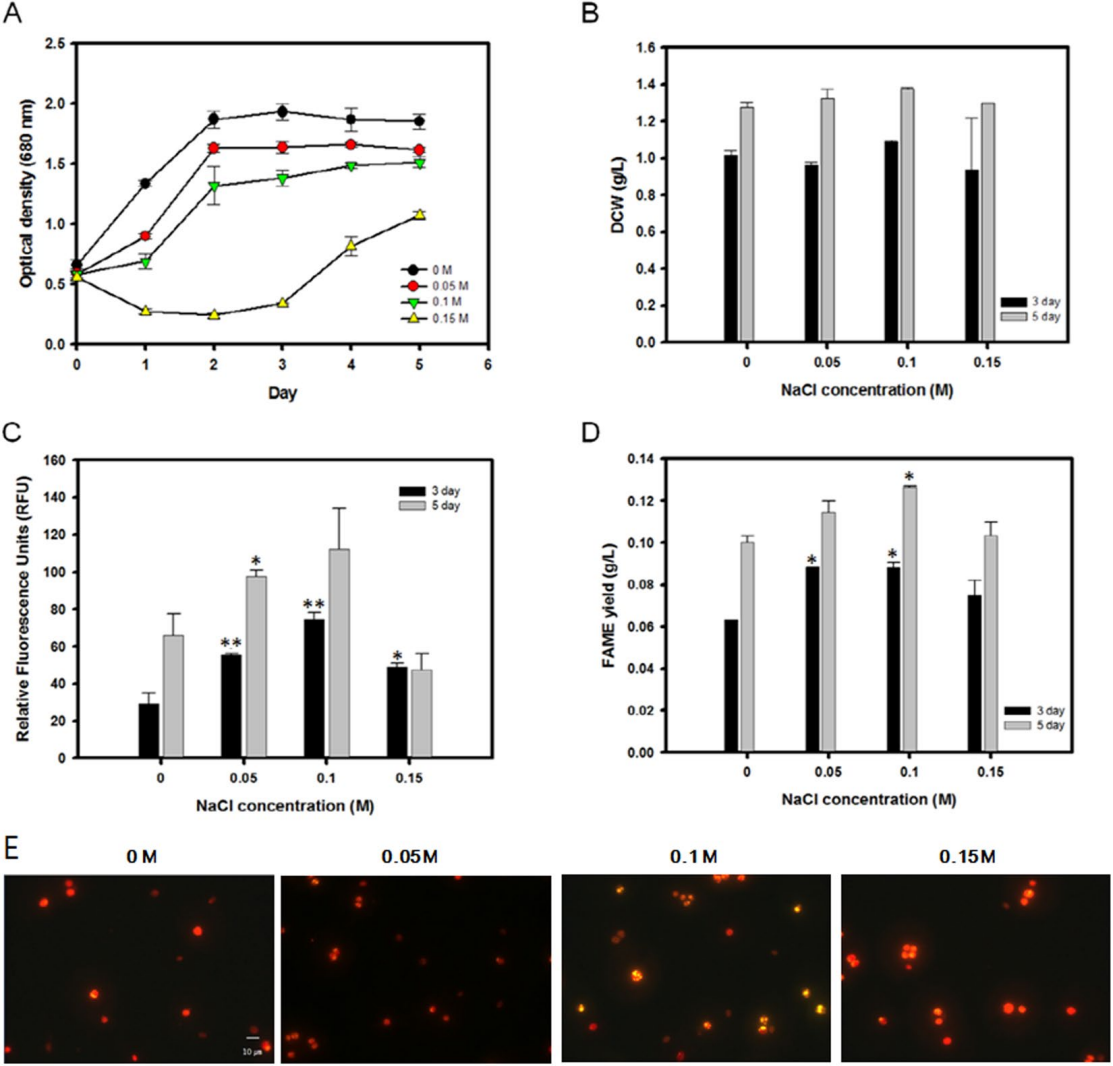
Cell proliferation and lipid accumulation of *C*. *reinhardtii* under various levels of osmotic stress. (**A**) Time course of cell growth (O.D. 680 nm). (**B**) Total dry cell weight at 3 and 5 days after osmotic stress induction. (**C**) Nile red fluorescence measurement at 3 and 5 days after osmotic stress induction. (**D**) Total lipid yield measured by *in situ* transesterification into FAME followed by gas chromatography. (**E**) Nile Red fluorescence microscopy of the cells at 0, 0.05, 0.10, and 0.15 M concentrations of NaCl. Data are represented as mean ± SD (*n* = 3). Significant differences were determined by Student’s t-test and indicated by asterisks (*P < 0.05, **P < 0.01, ***P < 0.001).

To precisely determine the effects of various levels of osmotic stress on *C*. *reinhardtii*, the microalgae were cultivated in TAP medium containing various concentrations of NaCl ranging from 0 to 0.15 M. *C*. *reinhardtii* was initially grown in traditional TAP medium without NaCl for 3 days to facilitate normal growth. On day 3, the cells at the mid-exponential stage were transferred to an osmotic stress environment with various concentrations of NaCl. The cells subsequently reached the stationary phase of growth within 2 days after the transfer, except for the cells treated with 0.15 M NaCl which resulted in a substantial growth defect (Fig. 2A).

To examine the influence of osmotic stress on neutral lipid production, the cells were stained with Nile red on days 3 and 5. There was an overall positive correlation between the total neutral lipid within the culture and the osmotic stress for both days 3 and 5 (Figs 2C, E and S2). As the NaCl content was increased from 0 to 0.1 M, the fluorescence measurement on day 5 increased by 1.7-fold. However, excessive osmotic stress at 0.15 M NaCl led to a severe decline in the fluorescence intensity, most likely due to a decrease in the cell viability. The fluorescence measurement showed an identical trend on day 3 as well, albeit at lower intensity levels. In addition to the Nile red fluorescence measurements, precise quantification of all aliphatic lipids within the biomass was achieved by direct conversion of the lipids within the harvested biomass to FAME using the modified Folsch’s method followed by gas chromatography. The total esterifiable lipid yields showed similar trends compared to that of the Nile red measurement (Fig. 2D). The total lipid yield on day 5 peaked at 0.13 g/L at a 0.1 M NaCl concentration up from 0.1 g/L under a zero stress environment. Likewise, the total lipid content within the biomass increased from 7.87% to 9.19% over the same range of salinity (Table 1).

**Table 1:** Total dry cell weight, lipid contents, and lipid yields of *C*.*reinhardtii* under various levels of osmotic stress.

NaCl	DCW (g/L)		FAME contents (% w/w)		FAME yield (g/L)	
Conc. (M)	3 day	5 day	3 day	5 day	3 day	5 day
0	1.02 ± 0.02	1.28 ± 0.03	6.19 ± 0.12	7.87 ± 0.02	0.06 ± 0.01	0.10 ± 0.01
0.05	0.96 ± 0.02	1.33 ± 0.05	9.16 ± 0.17	8.66 ± 0.06*	0.08 ± 0.01*	0.11 ± 0.01
0.10	1.09 ± 0.01	1.38 ± 0.01	8.08 ± 0.17	9.19 ± 0.03*	0.09 ± 0.01*	0.13 ± 0.01
0.15	0.94 ± 0.28	1.30 ± 0.01	7.98 ± 1.76	7.95 ± 0.47	0.07 ± 0.04	0.10 ± 0.04

Data are expressed as mean ± SD (*n* = 3). Significant differences, as determined by Student’s t-test, are indicated by an asterisk (*P < 0.05, **P < 0.01, ***P < 0.001).

### PD98059 mediated inhibition of MAPK suppresses lipid productivity under osmotic stress

MEK inhibitor PD98059 was added to the culture medium at varying concentrations while the NaCl concentration was fixed at 0.1 M. This was due to the fact that the 0.1 M NaCl concentration exhibited the highest levels of neutral lipid productivity without causing excessive inhibitory effects on cellular growth (Fig. 2). *C*. *reinhardtii* was grown in salt-free TAP medium for 3 days until the mid-exponential stage of growth before being transferred to the saline stress medium with PD98059.

**Figure 3:**
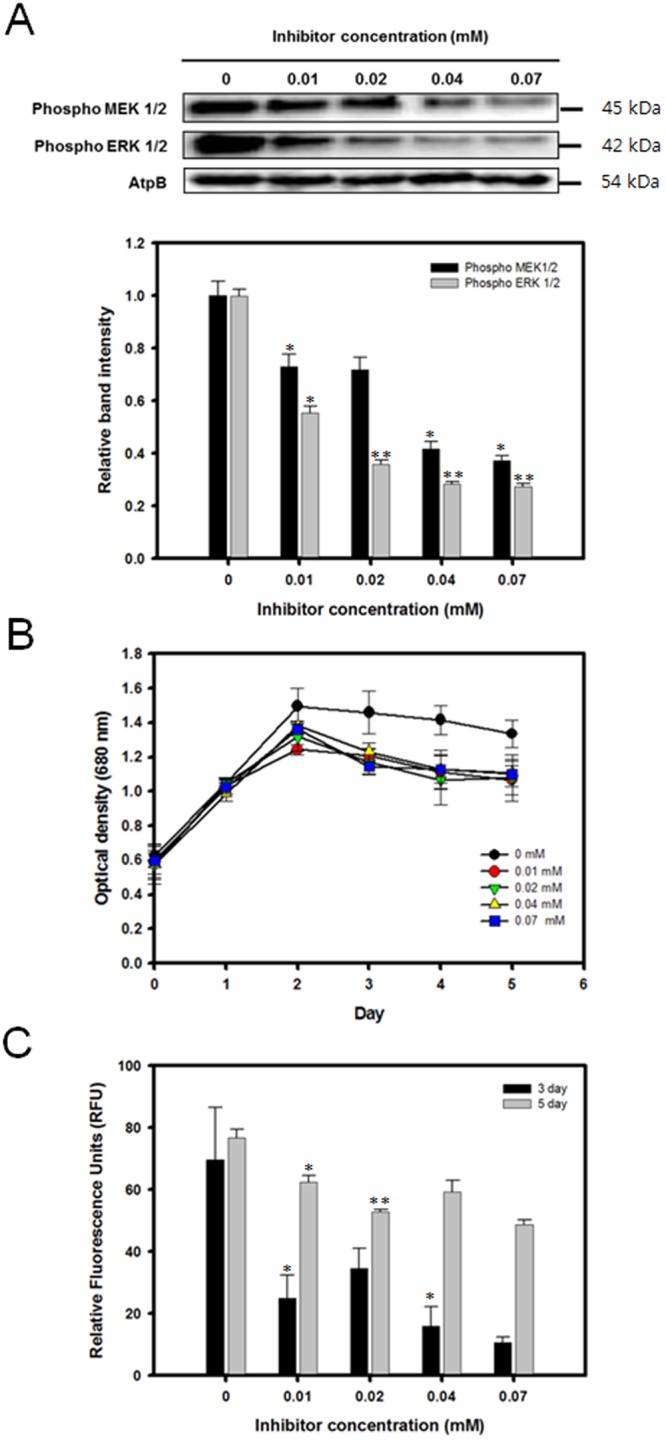
Expression level of MEK-ERK, cell growth profile, and lipid accumulation of *C*. *reinhardtii* grown in media containing 0.1 M NaCl and various concentrations of the MEK inhibitor PD98059. (**A**) Western blot analysis against anti-MEK1/2 and ERK1/2. ATPβ antibody was detected as a loading control. Full-length blots are presented in Supplementary Fig. S6. (**B**) Cell growth (O.D. 680 nm). (**C**) Nile red fluorescence measurement at 3 and 5 days after osmotic stress induction. Data are represented as mean ± SD (n = 3). Significant differences were determined by Student’s t-test and indicated by asterisks (*P < 0.05, **P < 0.01, ***P < 0.001).

Because it is important to confirm the dose dependent effects of PD98059 on the downregulation of MAP kinase in *C*. *reinhardtii*, western blot was done to evaluate the expression of both MEK1/2 and ERK1/2 for increasing concentrations of the inhibitor. For the western blot analysis, 1.5 million cells were prepared and blotted for the MEK1/2 and ERK1/2 activities using the same antibodies and protocol as above. It was observed that the band intensity at 45 kD corresponding to MEK1/2 and at 42 kD corresponding to ERK1/2 became weaker as the inhibitor concentration was increased (Fig. 3A). The band intensity for MEK1/2 fell by 27.02% even with the 0.01 mM inhibitor concentration, and a further decrease of 62.73% was observed when the inhibitor concentration was increased up to 0.07 mM. As for ERK1/2, the band intensity decreased by up to 72.65% in the presence of 0.07 mM of the MEK1/2 inhibitor. The above results indicate that PD98059 successfully inhibited the phosphorylation of MEK1/2, which in turn also reduced the activity of the downstream ERK1/2.

Next, both the cellular growth and lipid accumulation profile of *C*. *reinhardtii* in the presence of the MEK inhibitor was measured (Figs 3B, C, and S3). The cells reached the stationary phase of growth 2 days after being transferred to the TAP medium containing NaCl and PD98059. Although the culture without the inhibitor had the highest optical density, the growth rate did not further decrease under higher concentrations of PD98059. (Fig. 3B). In addition, the total dried cell weight (DCW) was measured twice at day 3 and 5 after the cells were transferred to the medium containing 0.1 g/L NaCl and 0.04 mM inhibitor. When the cells were subjected to the MEK inhibitor, a slight decrease in cellular growth was observed. Compared to the culture without inhibitor, the total DCW declined by 0.1 g/L by day 5 when the inhibitor concentration was 0.04 mM (Table 2).

**Table 2:** Total dry cell weight, lipid contents, and lipid yields of C.reinhardtii grown under various concentrations of the MEK inhibitor pd98059.

Inhibitor	DCW (g/L)		FAME contents (% w/w)		FAME yield (g/L)	
Conc.(mM)	3 day	5 day	3 day	5 day	3 day	5 day
0	0.9 ± 0.03	0.93 ± 0.09	9.97 ± 0.77	11.83 ± 0.07	0.09 ± 0.01	0.11 ± 0.01
0.04	0.8 ± 0.01*	0.83 ± 0.01	9.99 ± 1.12	8.42 ± 1.10*	0.08 ± 0.01	0.07 ± 0.01*

Data are expressed as mean ± SD (n = 3). Significant differences, as determined by Student’s t-test, are indicated by an asterisk (*P < 0.05, **P < 0.01, ***P < 0.001).

The ability of the cells to accumulate lipids was also found to be affected when MAPK activation was inhibited. Even under the lowest inhibitor concentration of just 0.01 mM, the relative fluorescence units declined by 64.22% at day 3 and by 18.71% at day 5 (Fig. 3C). Increasing the concentration of the MEK inhibitor appeared to further suppress the neutral lipid accumulation under osmotic stress. The lipids were measured by gas chromatography on day 3 and 5. At day 5, the FAME yield decreased from 0.11 g/L to 0.07 g/L with a 0.04 mM MEK inhibitor concentration (Table 2).

### Enhancement of MAPK activity promotes lipid productivity under osmotic stress

In addition to the MAPK suppression study, the microalgae were also grown in the presence of C6 ceramide, a known MAPK activator^30^. Like in the previous experiments, the cells were grown in normal TAP medium for 3 days before they were transferred to a 0.1 M NaCl medium containing from 0 to 0.07 mM C6 ceramide. The cells reached stationary phase 3 days after being transferred to the culture medium containing NaCl and the activator.

**Figure 4:**
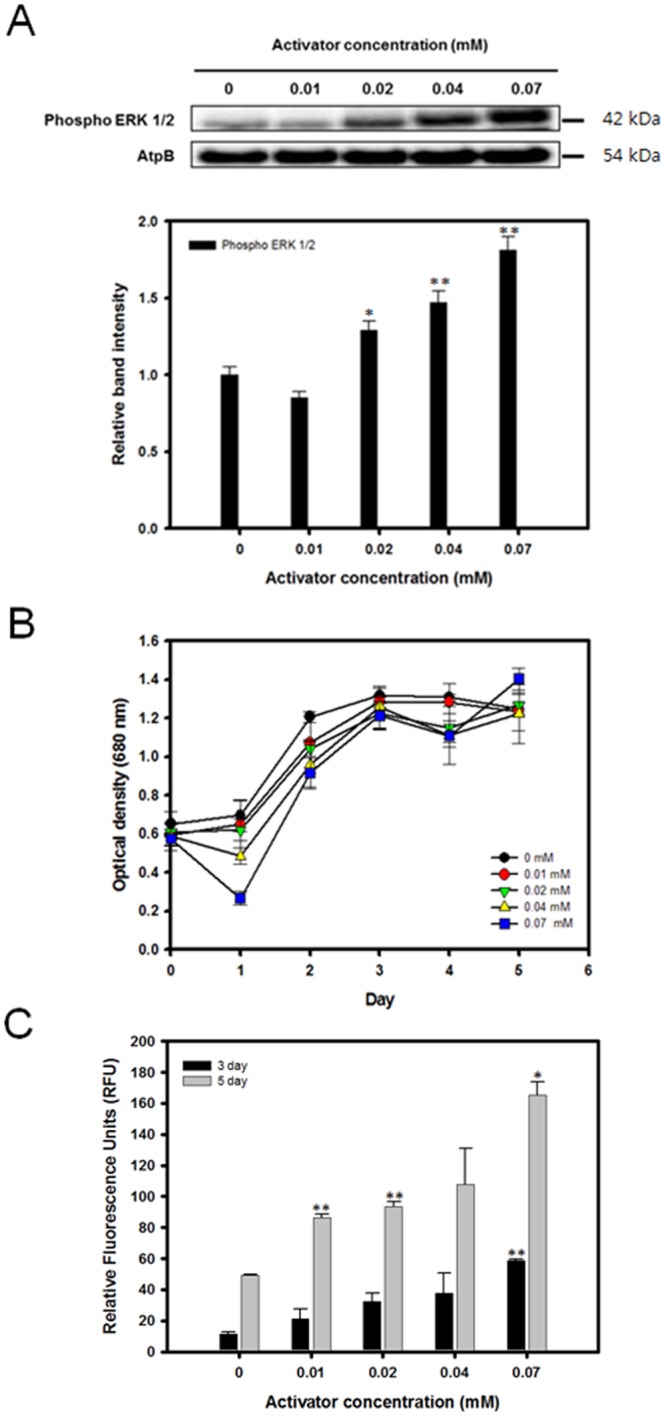
Expression level of ERK, cell growth profile, and lipid accumulation of *C*. *reinhardtii* grown in media containing 0.1 M NaCl with various concentrations of the ERK activator C6 ceramide. (**A**) Western blot analysis against anti-ERK1/2. ATPβ antibody was detected as a loading control. Full-length blots are presented in Supplementary Fig. S6. (**B**) Cell growth (O.D. 680 nm). (**C**) Nile red fluorescence measurement at 3 and 5 days after osmotic stress induction. Data are represented as mean ± SD (*n* = 3). Significant differences were determined by Student’s t-test and indicated by asterisks (*P < 0.05, **P < 0.01, ***P < 0.001).

To verify that the addition of C6 ceramide to the cultivation medium had a dose dependent activation of the MEK-ERK pathway, western blot analysis was done with the identical methods detailed in the previous experiments. However, in this case, only ERK1/2 was analyzed by western blot because C6 ceramide only activates the downstream ERK (MAPK) component of the pathway. The relative intensity of the 42 kDa band corresponding to phosphorylated ERK1/2 steadily increased under higher concentrations of the activator (Fig. 4A). The total relative band intensity increased up to 81.14% at 0.07 mM C6 ceramide.

The effects of C6 ceramide and the upregulated ERK activities on the growth and lipid accumulation were assessed with the same methods as described in the earlier sections. In general, the activator concentration did not appear to have a substantial influence on the overall growth of *C*. *reinhardtii* (Fig. 4B). The overall growth curve in terms of the optical density remained comparable when the activator concentration was increased from 0 to 0.07 mM. However, the total DCW exhibited a slightly increasing trend as the activator concentration was increased (Table 3). By day 5, an increase in the DCW from 0.88 g/L to 0.95 g/L was observed with the C6 ceramide concentration of 0.04 mM. The neutral lipid accumulation showed a positive correlation with the added activator concentration (Figs 4C and S4). By day 5, the Nile red fluorescence showed an increase when 0.07 mM of the activator was added (Fig. 4C). The total lipid yield and cellular lipid content also showed similar trends. As for the lipid accumulation, the FAME yield showed a moderate increase of up to 22.22% with the 0.04 mM activator concentration after 5 days (Table 3).

**Table 3:** Total dry cell weight, lipid contents, and lipid yields of *C*.*reinhardtii* grown under various concentrations of C6 ceramide.

Activator	DCW (g/L)		FAME contents (% w/w)		FAME yield (g/L)	
Conc.(mM)	3 day	5 day	3 day	5 day	3 day	5 day
0	0.77 ± 0.06	0.88 ± 0.05	10.35 ± 0.49	10.20 ± 0.56	0.08 ± 0.01	0.09 ± 0.01
0.04	0.78 ± 0.05*	0.95 ± 0.02	11.50 ± 0.54	11.56 ± 0.81	0.09 ± 0.01	0.11 ± 0.01

Data are expressed as mean ± SD (n = 3). Significant differences, as determined by Student’s t-test, are indicated by an asterisk (*P < 0.05, **P < 0.01, ***P < 0.001).

### JNK MAPK signaling is crucial for cell growth under osmotic stress environment

**Figure 5:**
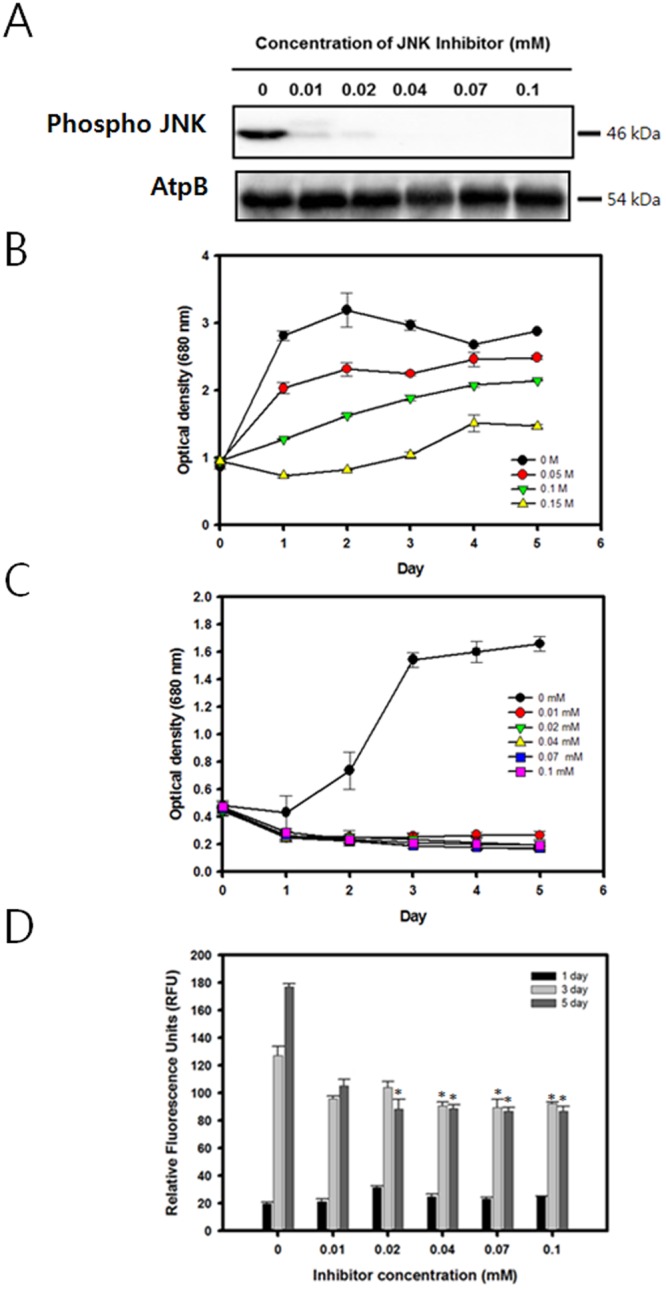
The expression level of JNK and cell growth profile and lipid accumulation of *C*. *reinhardtii* grown in media containing 0.1 M NaCl with various concentrations of the JNK inhibitor SP600125. (**A**) Western blot analysis against anti-JNK. Full-length blots are presented in Supplementary Fig. S6. (**B**) Cell growth (O.D. 680 nm) without SP600125 (**C**) Cell growth (O.D. 680 nm) with various concentrations of SP600125. (**D**) Nile red fluorescence measurement at 3 and 5 days after osmotic stress induction. Data are represented as mean ± SD (*n* = 3). Significant differences were determined by Student’s t-test and indicated by asterisks (*P < 0.05, **P < 0.01, ***P < 0.001).

The cells were grown in normal TAP medium for 3 days before transferring them to a saline growth environment containing various concentrations of SP600125, a known JNK inhibitor^31^. Similarly to the previous experiments, 0.1 M NaCl was chosen as an appropriate level of osmotic stress which does not cause an excessive reduction in cellular growth. To confirm the dose dependent effects of SP600125 on the expression of JNK, a western blot was performed. Protein samples from 1.5 million cells were prepared as in the previous experiments, and phospho-specific mammalian JNK antibodies were used for the detection. It was shown that SP600125 had a potent effect on the suppression of the JNK activity (Fig. 5A). Even at a 0.01 mM concentration, the relative band intensity fell by up to 86.63%. At higher concentrations of the inhibitor, the JNK activity was found to be negligible.

The effects of the JNK inhibitor on both the cellular growth and lipid biosynthesis in an osmotic stress environment were evaluated. Remarkably, the inhibition of the JNK pathway was found to dramatically restrict the cellular growth in a saline environment even at the lowest inhibitor concentration of 0.01 mM (Fig. 5B, C). The decrease in the growth was so severe that the biomass productivity was negligible for all cases except for the 0 mM inhibitor control. By day 5, the Nile red fluorescence measurement also showed a substantial decrease of 40.53% at the 0.1 mM inhibitor concentration (Fig. 5D).

## Discussion

In the present study, *C*. *reinhardtii* was investigated under various levels of osmotic stress and MAPK regulators. It has generally been established that subjecting microalgae to osmotic stress results in marked inhibition of growth as well as a moderate accumulation of lipid molecules^32^. As expected, increasing the concentrations of NaCl resulted in an overall reduction in cellular growth (Fig. 2A). The highest optical density (680 nm) was observed for the culture with 0 M NaCl, while the growth was substantially inhibited when the cells were transferred to the 0.15 M NaCl environment. This indicates that the cells lack the ability to proliferate under higher levels of salinity, particularly above 0.1~0.15 M range.

Interestingly, however, the biomass productivity in terms of the dry cell weight (DCW) did not decrease even at the highest levels of osmotic stress (Table 1) when the total DCW was measured on day 3 and 5 after the cells were subjected to osmotic stress (Fig. 2B). In fact, a slightly higher biomass productivity of 1.38 g/L was observed with 0.1 M NaCl compared to that of 1.28 g/L with the control. A similar discrepancy between the DCW yield and the growth curve was again observed when ERK1/2 was artificially upregulated with C6 ceramide. (Table 3) Discrepancies between the DCW and the optical density is sometimes caused by a possible presence of residual salts within the weight filters. However, this is unlikely as the cells have been washed with deionized water prior to filtration. Therefore, these findings suggest that subjecting *C*. *reinhardtii* to a salt stress condition during the mid-exponential phase of growth results in a decrease of the specific growth rate without affecting the total biomass productivity. The observed discrepancy between the OD and DCW yield can best be explained by the increase in the cell size due to disruptions in the cell cycle machinery. A factor that can influence the cell size is a change in the cell cycle progression. A previous study has shown that subjecting the model land plant *A*. *thaliana* to prolonged osmotic stress results in cell cycle arrest and endoreduplication, which results in an increase in ploidy as well as in cell size^33^. Overexpression of the ERK pathway, as it was further shown in the ERK1/2 overexpression experiment, may be partially responsible because it was shown that MAP kinases have been known to be involved in controlling cell cycles in yeast by modulating the CDK activity^34^.

The stress response triggered by high salinity has been shown to have various physiological effects on *C*. *reinhardtii* including an inhibited specific growth rate and increased lipid accumulation^16^. Because MAP kinases are known to be involved in directing cellular responses against a variety of environmental stresses, they were expected to be highly activated when *C*. *reinhardtii* was subjected to osmotic stress. In most eukaryotes including microalgae, the ERK (extracellular signal regulated kinase) family MAPKs are in turn phosphorylated by an upstream MAPKK, specifically MEK (MAPK ERK kinase)^35^. The western blot analysis showed that both MEK1/2 and ERK1/2 were increasingly phosphorylated under higher levels of osmotic stress. (Fig. 1) This confirms the enhanced phosphorylation based activation of both upstream and downstream components of the MEK-ERK pathway under osmotic stress conditions.

As it was shown, subjecting *C*. *reinhardtii* to osmotic stress resulted in the upregulation of the MEK-ERK pathway as well as an increase in cellular lipids. Unlike the well characterized role of the MAPK pathway in cell cycle and cell proliferation, the causal link between MAPKs and cellular lipid synthesis and metabolism has not yet been established except for few examples in higher plants^29^. However, MAPK signaling is known to regulate numerous transcription factors in eukaryotes^36^. It is highly likely that ERK activates a number of transcription factors which in turn upregulate the transcription of downstream lipid synthesis genes. The role of transcription factors in storage lipid accumulation has been widely established in higher plants. For example, WRI1 is an AP2 family transcription factor that is widely described as a key element that regulates seed oil production in *A*.*thaliana*^37^. The AP2 family transcription factor has been shown to be involved during the abiotic stress response in plants and is also known to be activated by the Ras-Ras-MEK-ERK signaling cascade^38,39^. A number of other transcription factors, such as GmDof4 and GmDof11, have also been found to be responsible for direct and indirect regulation of lipid metabolism^40^. Critically, a recent genome-wide study of *Nannochloropsis oceanica* and *Nannochloropsis gaditana* directly revealed numerous putative transcription factors that are believed to be involved in lipid biosynthesis, including the orthologs of WRI1 and GmDof^41^. These previous studies in addition to the findings in the present study imply that there are strong correlations between the MAPK signaling pathway activated by osmotic stress and lipid biosynthesis in the microalga *C*. *reinhardtii*.

It was shown that during the cultivation under osmotic stress, the cells not only showed a noticeable accumulation of lipids but also an increased activation of MEK1/2 and ERK1/2. However, it is yet unclear whether lipid accumulation is influenced by the activation of the MAPK pathway or if the two are unrelated. To determine whether MAPK activation mediates lipid accumulation under stress conditions, it was necessary to examine the lipid production pattern of microalgae under osmotic stress conditions while the MAPK pathway was artificially regulated. For this purpose, PD98059 was used as an inhibitory agent of the MAPK pathway. PD98059 is a potent MEK specific inhibitor *in vitro* and *in vivo*, which functions by binding to the inactive form of MEK and prevents its activation by upstream MAP3Ks^42^. Preventing MEK activation would also prevent the downstream activation of ERKs, and hence, the application of PD98059 would prevent the cells from activating their MAPK signaling cascade under osmotic stress conditions. For the activation of the MAPK, C6 ceramide, which activates the downstream ERK1/2 of the MEK pathway, was administered. Hence C6 ceramide was predicted to have an overall effect that is opposite to PD98059.

**Figure 6:**
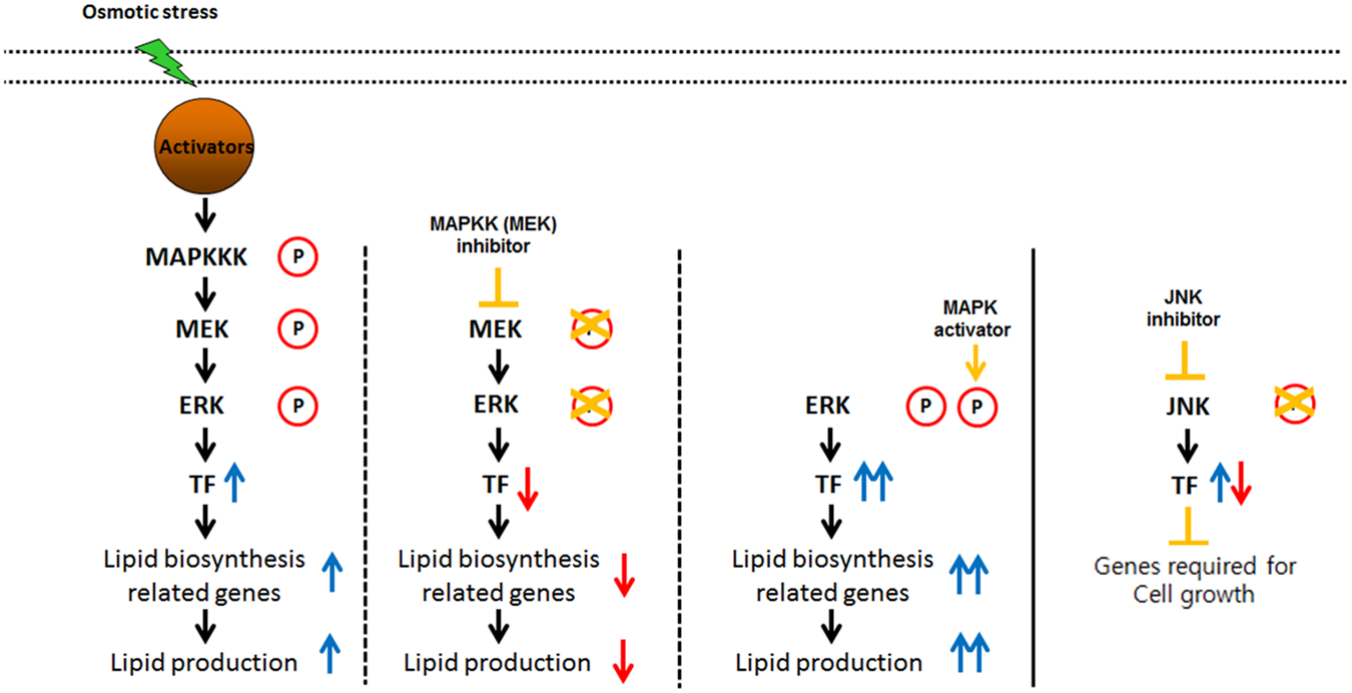
Proposed model of lipid biosynthesis and cell growth under osmotic stress.

As it was expected, the results of the ERK regulation experiments showed that the administration of PD98059 resulted in a decrease of lipid accumulation while C6 ceramide successfully upregulated the lipid productivity. This finding contradicts with a previous research which reported that ERK inhibition resulted in an increase in the overall lipid content^28^. However, in the previous study, ERK was inhibited under normal growth condition without any stress factors, while the current study subjected cells under osmotic stress. The difference in the results can be attributed to the fact that ERK activity under normal growth condition is rather low, and it is only highly activated under moderate to high level of osmotic stress, as shown in the western blot analysis (Fig. 1). The inhibitory effects of PD98059 on the MEK-ERK pathway likely resulted in a decrease of lipid accumulation despite the presence of osmotic stress by the downregulation of downstream transcription factors involved in lipid biosynthesis (Fig. 6). Likewise, enhancing the activity of the downstream ERK1/2 alone was able to increase the total lipid productivity. These results were directly opposite to the effects that were observed when the MEK-ERK pathway was suppressed, and it is shown that the activation by C6 ceramide resulted in a further enhancement of the lipid productivity. This finding further reinforces the proposition that the observed increase in lipid accumulation is likely caused by the upregulation of the ERK1/2 activity and the downstream transcription factors as shown by the treatment with C6 ceramide (Fig. 6).

In addition to the MEK-ERK pathway, the role of the JNK signaling pathway during osmotic stress was also examined. Like the ERK, it has been reported that the JNK pathway is also activated by external stress factors including irradiation, heat, and osmotic stress^43–45^. Since meaningful lipid productivity data could not be measured due to excessively low cell growth, the experiment was repeated at much lower concentrations of SP600125 from 0.001 to 0.05 mM (Fig. S5). The cells exhibited dose dependent recovery in growth rate under lower levels of JNK inhibitor. However, the cells still contained dramatically reduced lipid content even under the lowest concentration, which hints that the JNK pathway is positively correlated with the lipid induction. Also from these results, it can be concluded that the JNK dependent MAPK signaling pathway has a substantial effect on the cell growth of *C*. *reinhardtii* in an osmotic stress environment (Fig. 6). This coincides well with the conventional wisdom that the JNK MAP kinase is widely involved in apoptosis by regulating the NF-κB pathway^46^.

## Conclusion

In the present study, *C*. *reinhardtii* was investigated under various levels of osmotic stress and a MAPK inhibitor and activator. Osmotic stress was positively correlated with the ERK activity while the JNK pathway was inhibited. Further downregulation of ERK by PD98059 resulted in a decrease in the lipid yield while ERK upregulation had an opposite effect. On the other hand, inhibition of JNK under osmotic stress resulted in a drastic decrease in cell viability. These findings lead to the conclusion that MAPKs may have a crucial role in lipid biosynthesis and cell growth under osmotic stress through the regulation of a number of downstream genes.

## Methods

### Microalgal strain and cultivation parameters

Wild type *C*.*reinhardtii* (CC-124) cultures were cultivated with tris-acetate-phosphate (TAP) medium with agitation of 200 rpm at 25 °C, under constant illumination (130 μmol m^−2^ s^−1^). Initially, the seed culture was inoculated directly from the plate and was cultivated in 250 mL flasks for 3 days under normal condition without osmotic stress. For the induction of osmotic stress, the seed culture was harvested by centrifugation at 4000 rpm for 10 minutes and transferred to 24 well plate containing modified TAP medium with 0 to 0.15 M of NaCl. For the ERK inhibition study, the cells were transferred to a new TAP medium containing 0.10 M NaCl and 0 to 0.07 mM of PD98059 (Abcam, Cambridge, MA, USA). For the ERK activation study, the cells were transferred to a new TAP medium containing 0.10 M NaCl and 0 to 0.07 mM of C6 ceramide (Abcam, Cambridge, MA, USA). For the JNK inhibition study, the cells were transferred to a new TAP medium containing 0.10 M NaCl and 0 to 0.1 mM of SP600125.

### Growth and dry cell weight measurement

Cell growth was determined by measuring the optical density (OD) and dry cell weight (DCW). OD_680_ was measured with a UV/Visible spectrometer (Beckman Coulter, USA). The DCW was estimated by harvesting 5 mL of culture on the 3rd and 5th day after the transfer to the osmotic stress environment. The cells were filtered with GF/C filter paper (Whatman, USA) after washing with deionized water. DCW was estimated by weighing on a microbalance (CP224S, Sartorius, Germany) after drying at 105 °C overnight. Experiments were performed in triplicate.

### Neutral lipid measurement

Neutral lipids were analyzed by staining with Nile red fluorescent dye. Nile red stock solution was prepared at 0.1 mg mL-1 dissolved in DMSO. The Nile red analysis was done by staining 200 mL of cells with 5 mg/L Nile red (9- diethylamino -5H- benzo [alpha] phenoxazine - 5-one). After 30 minutes of a dark incubation period at 37 °C, Nile red fluorescence was measured using a fluorescence spectrophotometer (SpectraMax M2, Molecular Devices, USA) with an excitation and emission wavelength of 530 and 575 nm, respectively, following previously described protocols^47^. In order to complement the Nile Red fluorescence data, the neutral lipids within the sample were also quantified via colorimetric analysis using a triacylglyceride quantification kit (BioVision, Milpitas, CA, USA).

### Analysis of total esterifiable lipids by gas chromatography

*C*. *reinhardtii* culture was harvested and centrifuged at 4000 rpm for 10 minutes and washed with PBS. The frozen cell pellets were lyophilized at −50 °C. A known weight (within the range of 10~20 mg) of lyophilized microalgae underwent lipid extraction using 2 mL of chloroform:methanol (2:1, v/v) using Folch’s method with modifications^48^. The total esterifiable lipids within the cells were then converted into fatty acid methyl esters (FAMEs), by *in situ* esterification through the addition of 1 mL of methanol and 0.3 mL of sulfuric acid. The chloroform fraction was phase separated with 1 mL of deionized water and filtered with a 0.22 µm syringe filter. The FAMEs were analyzed by a gas chromatograph (GC) (HP5890; Agilent, CA) equipped with a flame ionization detector (FID) and a capillary column (Agilent, CA). The FAMEs were quantified by the addition of 0.5 mg of C17:0 internal standards (Sigma Aldrich) and comparing the peak areas. GC analyses were performed in triplicates.

### Thin layer chromatography (TLC) analysis

About 5 mg of the lyophilized cells was mixed with chloroform: methanol: deionized water (2:2:1.24, v/v/v). The mixture was centrifuged at 3000 rpm for 10 minutes, and filtered with 0.22 µm syringe filter after re-separation with chloroform:methanol:deionized water (2:1:0.8, v/v/v). After evaporation, the sample was loaded on Silica gel TLC plate (Merck, Germany), and the plate was immersed with hexane:diethyl ether: acetic acid (80:30:1, v/v/v). After spraying on the plate with 1% (w/v) primuline (Sigma-Aldrich) in acetone: deionized water (4:1, v/v), separated bands were detected by using exposure in ChemiDoc system (Bio-Rad), following previously described protocols^47^.

### Western blot analysis

*C*. *reinhardtii* cells were harvested 5th day after the treatment for Western blot analysis. Approximately 1.5 × 10^6^ cells of *C*. *reinhardtii* were harvested by centrifugation at 4000 rpm for 10 minutes and washed with PBS. The cell number was quantified with a hematocytometer (INCYTO, Korea) under a microscope (Nikon TS100, Tokyo, Japan). The cells were resuspended in a 100 μL mixture of Laemmli lysis buffer, beta mercaptoethanol, protease inhibitor and phosphatase inhibitor (19:1:1:1, v/v/v/v). The mixture was then heated at 100 °C for 30 min. for protein extraction. The supernatant was recovered by centrifuging the cells at 13000 RPM for 5 minutes. The protein lysate was run on a SDS page gel, which was subsequently electroblotted to a PVDF (polyvinylidene fluoride) membrane with the trans-blot turbo semi-dry transfer system (Bio-Rad). Immunodetection for the activation of ERK1/2 and MEK1/2 was done with phospho-p44/42 MAPK (Thr202/Tyr204) (Cell Signaling Technology, Beverly, MA, USA)^49^ and phospho-MEK1/2 (Ser217/221)^50^ antibodies. JNK was detected with the phospho-SAPK/JNK antibody (Cell Signaling Technology, Beverly, MA, USA)^51^. β-subunit of ATP synthase (ATPβ) antibody was used as an experimental loading control^52^. Signals were detected by using exposure in ChemiDoc system (Bio-Rad) with enhanced chemiluminescence substrate (Bio-Rad, USA).

## Electronic supplementary material

Supplementary Information
