# Supplementary material for: MAPK/ERK and JNK pathways regulate lipid synthesis and cell growth of Chlamydomonas reinhardtii under osmotic stress, respectively
Source: Sci Rep. 2018 Sep 14;8:13857. doi: 10.1038/s41598-018-32216-5 (PMC6138697; doi:10.1038/s41598-018-32216-5)

**Supplementary information**

**MAPK/ERK and JNK pathways regulate lipid synthesis and cell growth of *Chlamydomonas reinhardtii* under osmotic stress, respectively**

Ahreum Yang^a^, William I. Suh^b^, Nam Kyu Kang ^b^, Bongsoo Lee^a,^*,Yong Keun Chang^a,b,^*

*^a^Department of Chemical and Biomolecular Engineering, KAIST, 291 Daehak-ro, Yuseong-gu, Daejeon, 305-701, Republic of Korea*

*^b^Advanced Biomass R&D Center, KAIST, 291 Daehak-ro, Yuseong-gu, Daejeon, 305-701, Republic of Korea*

*Corresponding authors:

Bongsoo Lee

Tel: +82 42 350 5964; Fax: +82 42 860 3910

E-mail address: [bongsoolee@kaist.ac.kr](mailto:bongsoolee@kaist.ac.kr)

Yong Keun Chang

Tel: +82 42 350 3927; Fax: +82 42 860 3910

E-mail address: [changyk@kaist.ac.kr](mailto:changyk@kaist.ac.kr)

**Figure S1.** Phosphorylation of MAP kinases of *Chlamydomonas reinhardtii* cultivated under 0, 0.05, 0.1, and 0.15 M concentrations of NaCl were analyzed with phospho-specific antibodies. (A) Western blot analysis of ERK1/2 with relative band intensities. (B) Western blot analysis of JNK with relative band intensities. (C) Western blot analysis of p38 with relative band intensities.


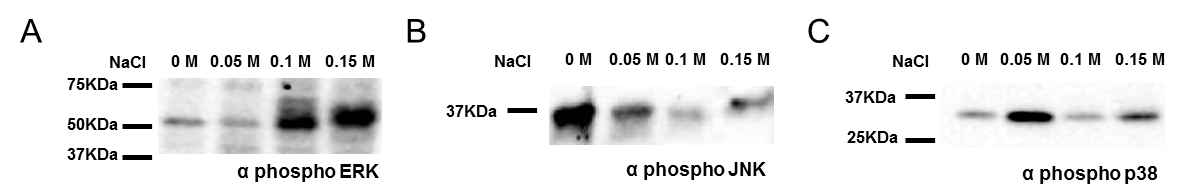


**Figure S2.** Neutral lipid accumulation of *C. reinhardtii* cultivated under 0, 0.05, 0.1, and 0.15 M concentrations of NaCl. (A) Neutral lipid measurement via TAG analysis (B) Thin layer chromatography (TLC) measurement with relative band intensities. (C) A representative image of TLC measurement at 5 days after osmotic stress induction.

##
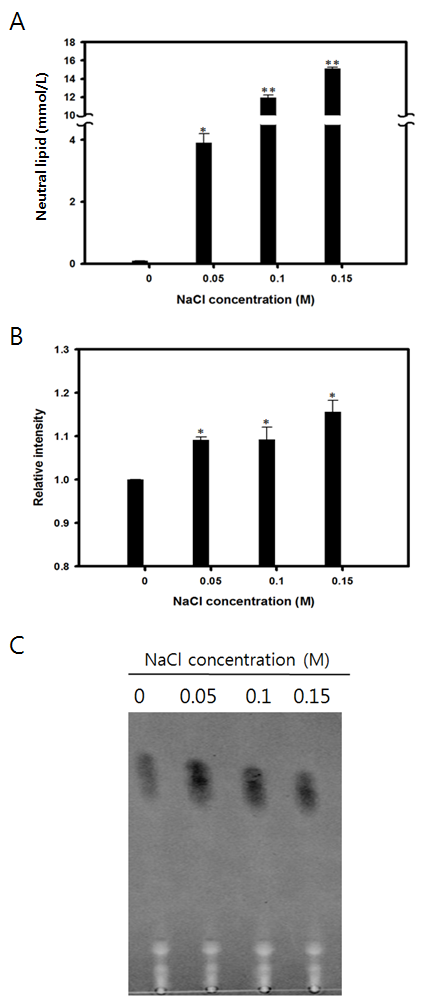


**Figure S3.** Neutral lipid accumulation of *C. reinhardtii* with 0.1 M NaCl and various concentrations of the MEK inhibitor PD98059. (A) Neutral lipid measurement via TAG analysis (B) Thin layer chromatography (TLC) measurement with relative band intensities. (C) A representative image of TLC measurement at 5 days after osmotic stress induction. (D) Nile Red fluorescence microscopy.


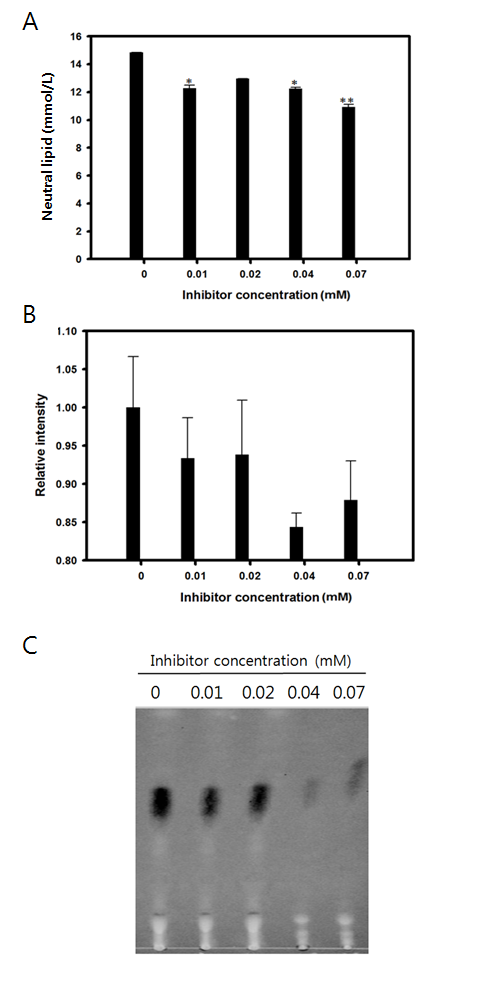


**
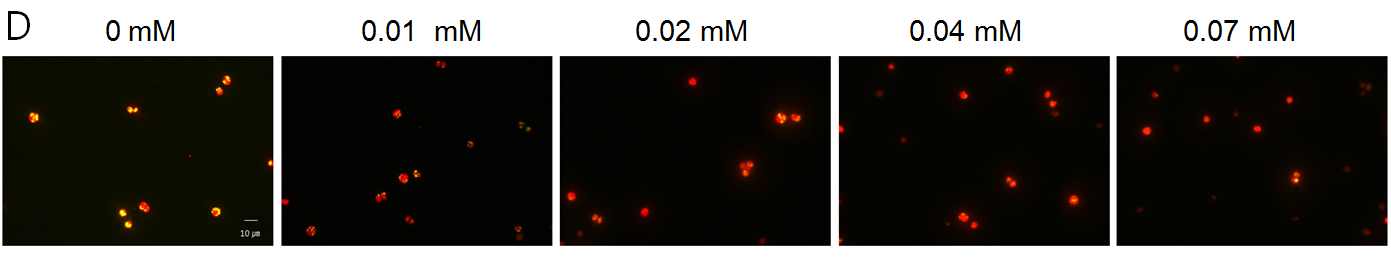
**

**Figure S4.** Neutral lipid accumulation of *C. reinhardtii* with 0.1 M NaCl and various concentrations of the ERK activator C6 ceramide. (A) Neutral lipid measurement via TAG analysis (B) Thin layer chromatography (TLC) measurement with relative band intensities. (C) A representative image of TLC measurement at 5 days after osmotic stress induction. (D) Nile Red fluorescence microscopy.


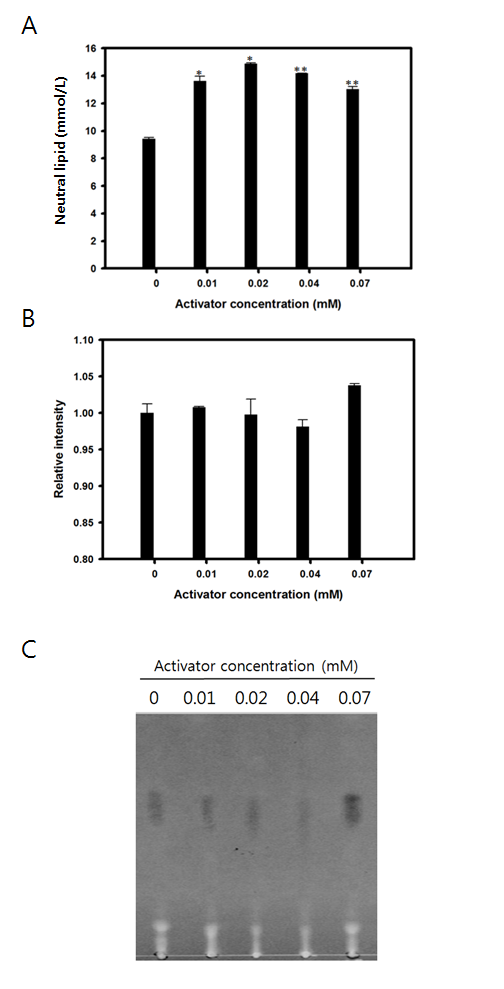


**
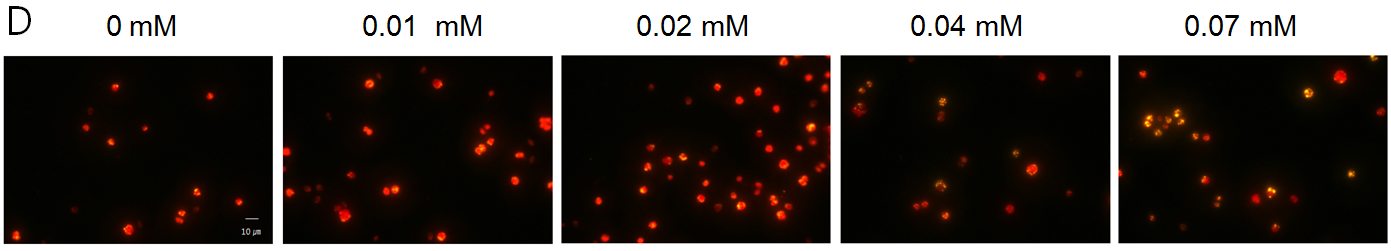
**

**Figure S5.** Cell growth profile and lipid accumulation of *C. reinhardtii* grown in media containing 0.1 M NaCl with various concentrations of JNK inhibitor. (A) Cell growth (O.D. 680 nm) with various concentrations of SP600125. (B) Nile red fluorescence measurement at 3 and 5 days after osmotic stress induction. (C) Nile Red fluorescence microscopy.


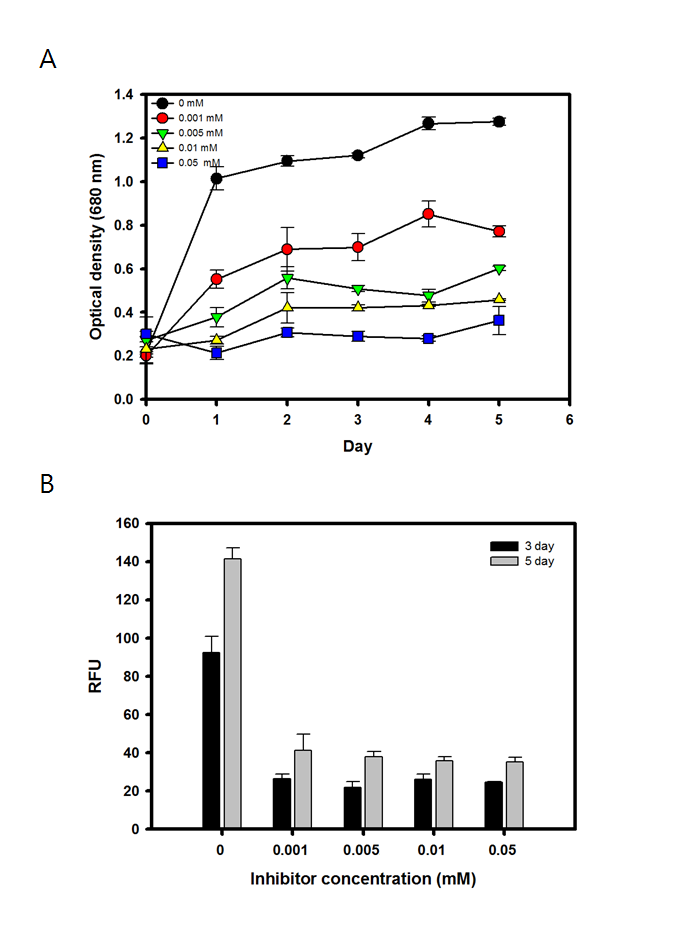

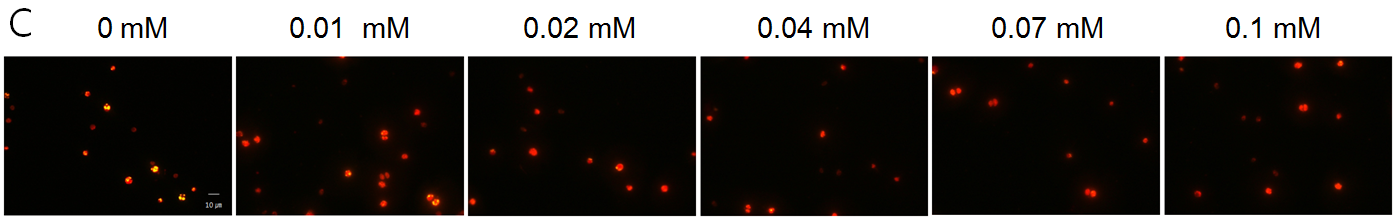


**Figure S6.** Uncropped full-length images of western blot experiments.


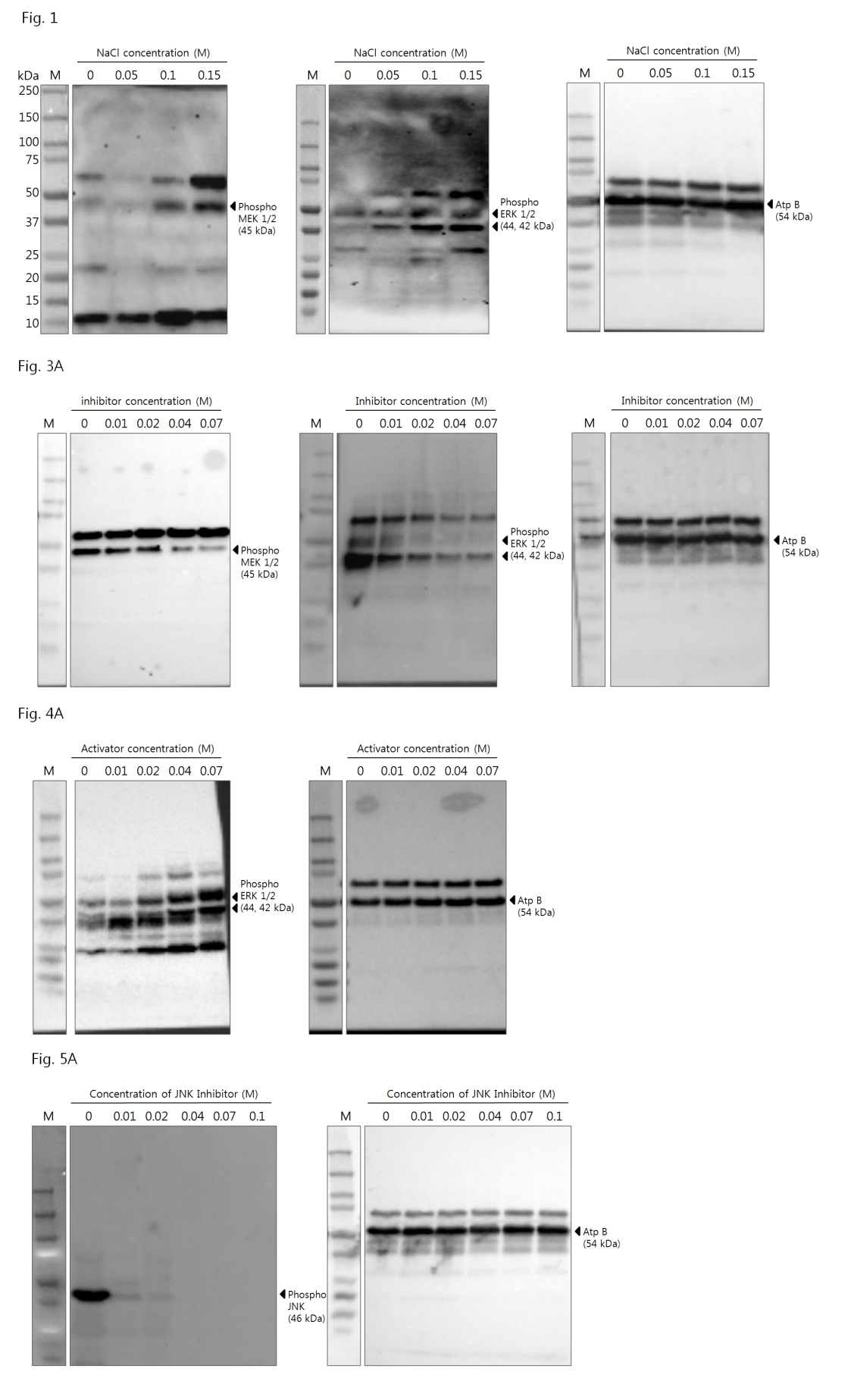

Supplement: Supplementary file 1 — Supplementary Information [file 41598_2018_32216_MOESM1_ESM.docx]
